# Estimates of the Association of Dementia With US Mortality Levels Using Linked Survey and Mortality Records

**DOI:** 10.1001/jamaneurol.2020.2831

**Published:** 2020-08-24

**Authors:** Andrew C. Stokes, Jordan Weiss, Dielle J. Lundberg, Wubin Xie, Jung Ki Kim, Samuel H. Preston, Eileen M. Crimmins

**Affiliations:** 1 Department of Global Health, Boston University School of Public Health, Boston, Massachusetts; 2 Population Studies Center, University of Pennsylvania, Philadelphia; 3 Department of Demography, University of California, Berkeley; 4 Leonard Davis School of Gerontology, University of Southern California, Los Angeles

## Abstract

**Importance:**

Vital statistics are the primary source of data used to understand the mortality burden of dementia in the US, despite evidence that dementia is underreported on death certificates. Alternative estimates, drawing on population-based samples, are needed.

**Objective:**

To estimate the percentage of deaths attributable to dementia in the US.

**Design, Setting, and Participants:**

A prospective cohort study of the Health and Retirement Study of noninstitutionalized US individuals with baseline exposure assessment in 2000 and follow-up through 2009 was conducted. Data were analyzed from November 2018 to May 2020. The sample was drawn from 7489 adults aged 70 to 99 years interviewed directly or by proxy. Ninety participants with missing covariates or sample weights and 57 participants lost to follow-up were excluded. The final analytic sample included 7342 adults.

**Exposure:**

Dementia and cognitive impairment without dementia (CIND) were identified at baseline using Health and Retirement Study self- or proxy-reported cognitive measures and the validated Langa-Weir score cutoff.

**Main Outcomes and Measures:**

Hazard ratios relating dementia and CIND status to all-cause mortality were estimated using Cox proportional hazards regression models, accounting for covariates, and were used to calculate population-attributable fractions. Results were compared with information on cause of death from death certificates.

**Results:**

Of the 7342 total sample, 4348 participants (60.3%) were women. At baseline, 4533 individuals (64.0%) were between ages 70 and 79 years, 2393 individuals (31.0%) were between 80 and 89 years, and 416 individuals (5.0%) were between 90 and 99 years; percentages were weighted. The percentage of deaths attributable to dementia was 13.6% (95% CI, 12.2%-15.0%) between 2000 and 2009. The mortality burden of dementia was significantly higher among non-Hispanic Black participants (24.7%; 95% CI, 17.3-31.4) than non-Hispanic White participants (12.2%; 95% CI, 10.7-13.6) and among adults with less than a high school education (16.2%; 95% CI, 13.2%-19.0%) compared with those with a college education (9.8%; 95% CI, 7.0%-12.5%). Underlying cause of death recorded on death certificates (5.0%; 95% CI, 4.3%-5.8%) underestimated the contribution of dementia to US mortality by a factor of 2.7. Incorporating deaths attributable to CIND revealed an even greater underestimation.

**Conclusions and Relevance:**

The findings of this study suggest that the mortality burden associated with dementia is underestimated using vital statistics, especially when considering CIND in addition to dementia.

## Key Points

**Question:**

Is the association of dementia with national mortality levels underestimated by death certificate data?

**Findings:**

In this nationally representative cohort study of 7342 older adults, the estimated percentage of deaths attributable to dementia was 2.7 times that calculated from vital statistics data on the underlying cause of death.

**Meaning:**

The findings of this study suggest that dementia may represent a more important factor in US mortality than indicated by routine mortality statistics, highlighting the need to expand population-based interventions focused on dementia prevention and care.

## Teaser

This cohort study examines the reporting of dementia on death certificates in the US and the mortality burden associated with dementia.

## Introduction

Alzheimer disease and related dementias (ADRD) affect millions of individuals in the US and represent a major source of disease burden and health care costs in the US.^1,2^ An estimated 5.6 million adults in the US 65 years or older lived with ADRD in 2019.^3^ There are several types of dementia, including Alzheimer disease (AD), which accounts for 80% of the cases,^4^ vascular dementia, which accounts for an estimated 10% of the cases,^4^ and mixed cause, in which patients show signs of both AD and vascular dementia (approximately 50% of AD cases).^5^ In addition to ADRD, an estimated 18.8% of individuals in the US 65 years or older live with cognitive impairment without dementia (CIND),^6^ about a third of whom may develop ADRD within 5 years.^1^

The prevalence of ADRD and CIND increases rapidly with age and shows large racial inequities, with non-Hispanic Black individuals having about 3 times the prevalence of ADRD compared with non-Hispanic White individuals.^7^ In addition, ADRD is a major risk factor for mortality, increasing the risk of death by a factor of 2.^8,9,10^ In 2017, ADRD was the third leading cause of death in the US and was listed as the underlying cause of death on 261 914 death certificates.^11^

Comparisons of vital statistics with other data sources suggest that physicians and medical examiners substantially underreport ADRD on death certificates. In a community-based, prospective epidemiologic study, only one-quarter of deaths in patients with dementia had AD listed on the death certificate.^12^ Another study with adjudicated records showed that dementia cases were often coded using one of several more immediate causes of death, such as pneumonia, sepsis, and cardiovascular disease.^13^

Underreporting of ADRD on the death certificate may be explained by several factors.^14^ First, individuals with ADRD who die typically have multiple comorbidities, complicating the identification of a single underlying cause.^15,16^ Second, cognitive impairment may reduce the ability of individuals to report symptoms and receive diagnosis.^13^ Stigma about dementia may also contribute to lack of diagnosis.^14^ Some studies estimate that ADRD is underdiagnosed in more than half of individuals with the disease.^1^ In addition, in cases in which individuals have received an ADRD diagnosis, underreporting may occur if the medical certifier is not aware of the diagnosis.

Several studies have examined potential underreporting of the mortality burden of ADRD on death certificates. One study extrapolated the annual number of deaths among older adults with ADRD using data from the Chicago Health and Aging Project combined with national population and mortality data.^17^ Another study used data from the Religious Orders Study, the Rush Memory and Aging Project, and the Chicago Health and Aging Project to estimate deaths associated with dementia using a population-attributable fraction (PAF).^18^ As noted by the authors, a limitation of both studies was that their estimates were extrapolated using input parameters generated from several nonrepresentative data sources with unclear generalizability to the US population.

In addition to issues of generalizability, prior studies have usually focused on ADRD without considering CIND.^8,9,10,17,18,19^ Several studies estimated hazard ratios (HRs) for mortality based on disease severity or continuous scores, such as the Mini-Mental State Examination, but did not carry out the additional step of extrapolating to the population level.^20,21^ Recognizing that mild cognitive impairment can later transition to dementia,^22,23,24^ not considering deaths associated with CIND could result in underestimating the dementia mortality burden.

In the present study, we used nationally representative survey data with validated measures of cognitive status from the Health and Retirement Study (HRS) and linked cause-of-death records to examine the association of dementia and CIND with all-cause mortality. We obtained estimates of the percentage of US deaths attributable to dementia and CIND and compared our estimates with information derived from death certificates. Our approach incorporated high-quality, survey-based estimates of dementia status, which mitigates the risk of measurement error related to underreporting of dementia on decedents’ mortality records.

## Methods

### Data Source and Participants

The HRS is a nationally representative, longitudinal cohort study of community-dwelling adults older than 50 years and their spouses or partners of any age.^25^ The HRS is unique in its tracking of participants as they transition into nursing homes and its collection of data by proxy for respondents who are unable to complete the survey. In addition, the HRS fielded a nationally representative study of cognitive health and dementia—the Aging, Demographics, and Memory Study—which has been used to develop and validate classification algorithms for assessing cognitive status in the full HRS sample.^26,27^ Subsequent studies have applied these definitions to estimate the prevalence of dementia and CIND in the US older adult population.^6,26,28,29,30^

The present study included adults sampled in the 2000 wave of HRS (baseline). The age range was restricted to 70 to 99 years at baseline as dementia/CIND cases in persons younger than 70 years were sparse and sample sizes of those older than 99 years were limited. Of 7489 adults with nonmissing data on cognitive status, those with missing covariates (n = 88), sample weights (n = 2), or who were lost to follow-up (n = 57) were excluded, resulting in a final analytic sample of 7342 older adults (eFigure 1 in the Supplement). Written informed consent was obtained from all HRS participants. Institutional review board approval for use of the restricted HRS files with deidentified data was granted through the University of Southern California. This study followed the Strengthening the Reporting of Observational Studies in Epidemiology (STROBE) reporting guideline for cohort studies.

### Classification of CIND and Dementia 

Prior studies have developed and validated multiple algorithmic classifications of CIND and dementia status in the HRS using demographic data, cognitive test scores, and physical functioning as predictors. For our primary analyses, we used the Langa-Weir score cutoff, which was previously validated to identify dementia status against the Aging, Demographics, and Memory Study dementia diagnosis (the standard).^26^ In the Langa-Weir method, cognitive scores for self-respondents are based on tests of immediate and delayed recall of 10 words (score 0-10 each, for a total of 20), a serial 7s task, and a backward counting task (score 0-2) to yield a score ranging from 0 to 27. For proxy respondents, cognitive scores are based on a proxy’s assessment of respondent’s memory (0, excellent; 1, very good; 2, good; 3, fair; and 4, poor), proxy’s assessment of respondent’s limitations in 5 instrumental activities of daily living (managing money, taking medication, preparing hot meals, using telephones, and shopping for groceries) (score 0-5); and the interviewer’s assessment of respondent’s difficulty completing the interview because of cognitive limitation (0, none; 1, some; and 2, prevents completion) to yield a score ranging from 0 to 11. Respondents’ cognitive scores are then classified using a 3-level dementia status variable as normal cognitive functioning (self: 12-27 or proxy: 0-2), CIND (self: 7-11 or proxy: 3-5), or dementia (self: 0-6 or proxy: 6-11).

We also classified dementia status using 4 additional algorithms. These methods were the Herzog-Wallace, Wu, Hurd, and modified Hurd algorithms, which were described and validated against the Aging, Demographics, and Memory Study dementia diagnosis elsewhere.^30,31^ Since some of these methods required dichotomous outcomes, we only considered dementia (no dementia or dementia) in our sensitivity analyses, rather than a 3-level outcome incorporating CIND.

### Covariates and Outcome

Covariates were measured at baseline and included age, sex, race/ethnicity, educational attainment, smoking status, self-reported disease diagnoses, and US Census division. Self-reported disease diagnoses were ascertained by asking respondents to report whether a medical practitioner had ever informed them of the condition.

The HRS data were linked with National Death Index records by the National Center for Health Statistics using an approach previously described.^32^ The HRS-derived estimates of mortality and life expectancy correspond closely with estimates derived from vital statistics.^33^ The HRS-linked records include underlying cause of death (the disease or injury that initiates the chain of events leading to death) and any mention of a condition or cause of death on the death certificate classified by *International Statistical Classification of Diseases, Version 10* (*ICD-10*) codes. Deaths attributed to ADRD included any dementia-related diagnosis (*ICD-10* codes F00-F03, G30, G31.0-G31.1, and R54) or AD (*ICD-10* codes F00 and G30). In this study, we compared our primary outcome, the percentage of deaths attributable to ADRD according to a PAF estimate, with the proportion of dementia-related deaths according to underlying causes and with any mention of dementia on death certificates.

### Statistical Analysis

We used Cox proportional hazards regression models to estimate HRs relating dementia and CIND status to all-cause mortality. The primary model was adjusted for age, sex, race/ethnicity, educational attainment, smoking status, indicator variables for a prior diagnosis of hypertension, diabetes, heart disease, and stroke, and US Census division. These characteristics were selected for their plausible associations with cognitive status and mortality.

We censored individuals after 10 years of follow-up to limit the amount of time between assessment of exposure and outcome. The validity of the proportional hazards assumption was examined by testing the slope of the Schoenfeld residuals by dementia and CIND status and by fitting a time-varying coefficients model using the tvc() option in Stata.^34^ Since we observed no deviation from proportionality in hazards, we proceeded with our analysis.

Next, we combined the adjusted HRs with prevalence estimates to calculate the percentage of deaths attributable to dementia using the PAF. The PAF represents the proportional reduction in mortality over a specified interval that would have occurred by eliminating dementia or CIND from the population while maintaining the distributions of other risk factors. We estimated the PAF using the following formula: ∑ *^k^_i_* _ = 0 _*pd_i_*[(*HR_i_* − 1)/*HR_i_*], where *pd_i_* refers to the proportion of decedents in dementia category *i* and *HR_i_* refers to the HR with respect to mortality for an individual in category *i*.^35,36^ We also added our PAF estimates for dementia and CIND to produce a combined PAF, hereafter referred to as PAF*. We replicated our PAF estimates within subgroups of interest using risk estimates from stratified regression analyses.

To assess for potential underreporting of dementia on death certificates, we compared the PAF estimates with the proportion of deaths in our sample with dementia listed as the underlying cause and the proportion of deaths with any mention of dementia listed on the death certificate. We then repeated the comparisons described above using PAF* to obtain estimates of the degree of underreporting when both CIND and dementia were considered. To maximize the comparability of estimates, we limited the assessment of underlying and multiple-cause data to the same sample inclusion criteria and period of prospective follow-up (2000-2009). In sensitivity analyses, we repeated the PAF calculation using dichotomous dementia status classified using the Langa-Weir method and all 4 alternative classification algorithms (Herzog-Wallace, Wu, Hurd, and modified Hurd) (eTable 3 in the Supplement).^30,31^ Data were analyzed from November 2018 to May 2020.

Analyses were carried out using Stata, version 14 (StataCorp), as well as SAS, version 9.4 (SAS Institute). Population-attributable fractions and their 95% CIs were estimated using the punafcc package.^37^ We included respondent-level sampling weights calculated by the HRS that adjust for the complex sampling design and nonresponse. Sample weighting and adjustment for clustering and stratification were made using the svy command in Stata. We estimated robust SEs to account for household clustering in the sample design.

## Results

**Figure 1.**
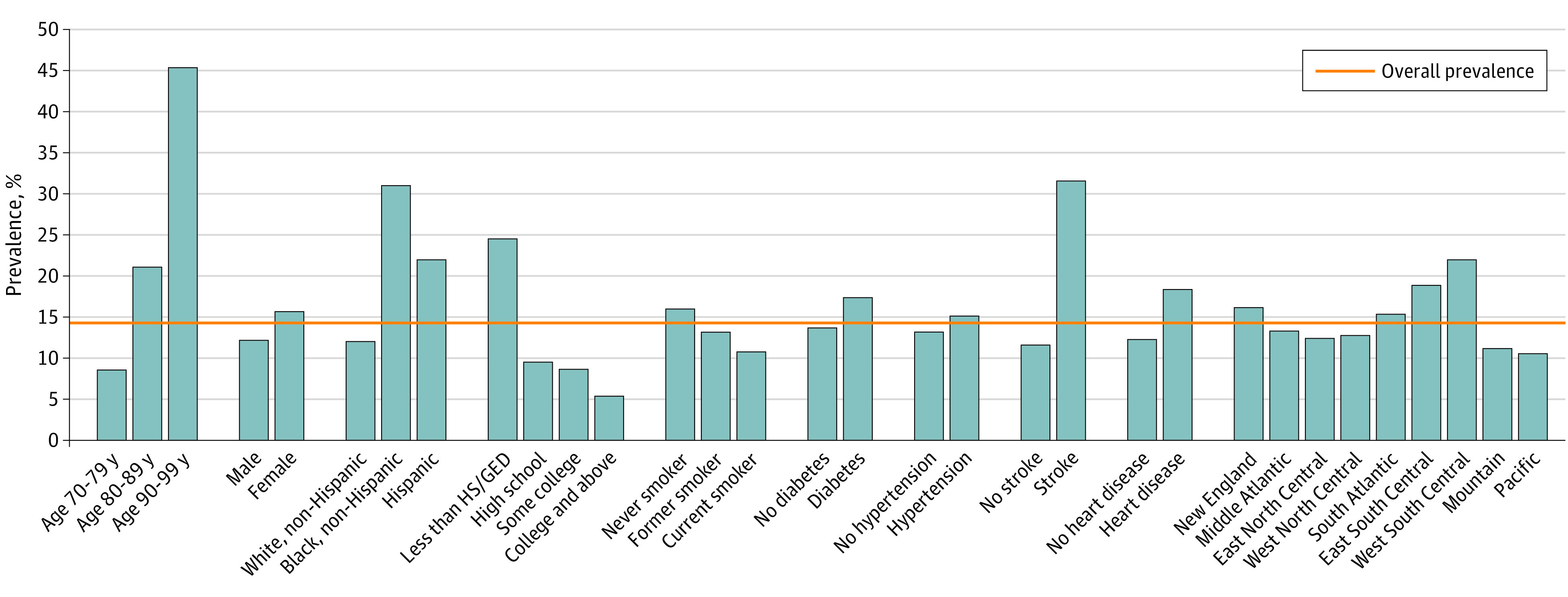
Prevalence of Dementia for Population and Subgroups Defined According to Sociodemographic Characteristics and Health Status HS/GED indicates high school/general educational development.

Table 1 reports the number and weighted percentages for baseline characteristics of the sample. Of the 7342 older adults, data on 1030 individuals (13.4%) were reported by proxy, 4348 individuals (60.3%) were women, 2994 individuals were men (39.7%), 4533 individuals (64.0%) were aged 70 to 79 years, 2393 individuals (31.0%) were aged 80 to 89 years, and 416 individuals (5.0%) were aged 90 to 99 years at baseline. The proportion of adults in the complete sample with dementia was 14.3%, and the proportion with CIND was 24.7%. Figure 1 and eTable 1 in the Supplement report the prevalence of dementia in the sample and among decedents. Overall, the prevalence of dementia (22.4%) and CIND (29.3%) was higher among decedents than in the complete sample.

**Table 1.** Descriptive Statistics of the Study Sample (N = 7342)

Characteristic	No. (%)^a^
Dementia	1133 (14.3)
CIND	1843 (24.7)
Interview type	
Self-reported	6312 (86.6)
Proxy-reported	1030 (13.4)
Age category, y	
70-79	4533 (64.0)
80-89	2393 (31.0)
90-99	416 (5.0)
Sex	
Male	2994 (39.7)
Female	4348 (60.3)
Race/ethnicity	
Non-Hispanic	
White	5920 (85.4)
Black	828 (8.1)
Other	125 (1.8)
Hispanic	469 (4.7)
Educational level	
<High school/GED	2826 (36.5)
High school	2245 (31.7)
Some college	1231 (17.0)
≥College	1040 (14.8)
Smoking status	
Never	3346 (45.8)
Former	3417 (46.2)
Current	579 (8.0)
Ever diagnosed with	
Diabetes	1168 (15.3)
Hypertension	3908 (53.1)
Stroke	978 (13.4)
Heart disease	2403 (32.6)
US Census division^b^	
New England	343 (5.7)
Middle Atlantic	965 (13.1)
East North Central	1265 (18.0)
West North Central	636 (9.5)
South Atlantic	1709 (19.5)
East South Central	334 (4.9)
West South Central	767 (10.0)
Mountain	346 (5.0)
Pacific	977 (14.3)

Abbreviations: CIND, cognitive impairment without dementia; GED, general educational development.

^a^ Weighted percentages and unweighted frequencies are presented.

^b^ New England (Connecticut, Maine, Massachusetts, New Hampshire, Rhode Island, and Vermont), Middle Atlantic (New York, New Jersey, and Pennsylvania), East North Central (Indiana, Illinois, Michigan, Ohio, and Wisconsin), West North Central (Iowa, Kansas, Minnesota, Missouri, Nebraska, North Dakota, and South Dakota), South Atlantic (Delaware; Washington, DC; Florida; Georgia; Maryland; North Carolina; South Carolina; Virginia; and West Virginia), East South Central (Alabama, Kentucky, Mississippi, and Tennessee), West South Central (Arkansas, Louisiana, Oklahoma, and Texas); Mountain (Arizona, Colorado, Idaho, New Mexico, Montana, Utah, Nevada, and Wyoming), Pacific (Alaska, California, Hawaii, Oregon, and Washington).

**Figure 2.**
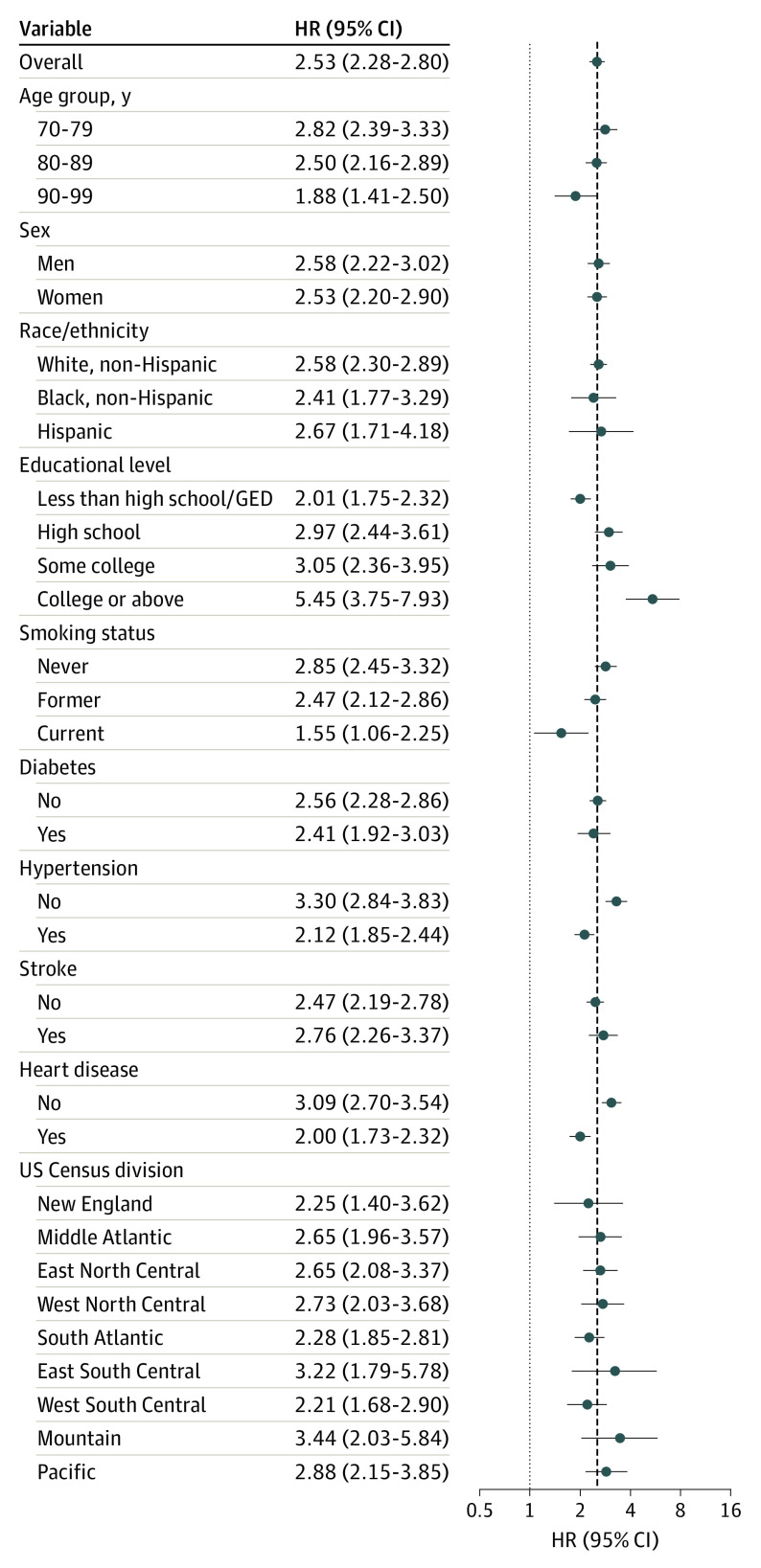
Mortality Risk Estimates for Dementia in Overall Population and by Subgroups Defined According to Sociodemographic Characteristics and Health Status GED indicates general educational development; HR, hazard ratio.

Figure 2 presents estimates of the association of dementia with mortality for the overall population and across subgroups. The corresponding Kaplan-Meier survival curve by cognitive status is presented in eFigure 2 in the Supplement. The HRs for dementia and CIND were relatively stable across sequential model adjustments (eTable 2 in the Supplement). In the fully adjusted model, the HRs were 2.53 (95% CI, 2.28-2.80) for dementia and 1.53 (95% CI, 1.41-1.67) for CIND.

An estimated 13.6% (95% CI, 12.2%-15.0%) of deaths were attributable to dementia (Table 2). The proportion of deaths assigned to dementia as an underlying cause on death certificates was 5.0% (95% CI, 4.3%-5.8%), suggesting underreporting by more than a factor of 2.7. Across subgroups examined, the highest mortality burden of dementia was observed in non-Hispanic Black older adults (24.7%; 95% CI, 17.3%-31.4%) compared with Hispanic (20.7%; 95% CI, 12.0%-28.5%) and non-Hispanic White (12.2%; 95% CI, 10.7%-13.6%) individuals (Table 2). The mortality burden of dementia was also significantly greater among individuals with less than a high school education (16.2%; 95% CI, 13.2%-19.0%) compared with those with a college education (9.8%; 95% CI, 7.0%-12.5%).

**Table 2.** Percentage of Deaths Attributable to Dementia Contrasting Underlying, Any Mention, and PAF-Based Estimates

Characteristic	Dementia, % (95% CI)		
	Underlying	Any mention^a^	PAF
All	5.0 (4.3-5.8)	16.4 (15.2-17.6)	13.6 (12.2-15.0)
Age groups, y			
70-79	4.1 (3.1-5.0)	12.2 (10.7-13.8)	10.2 (8.4-12.0)
80-89	5.6 (4.4-6.7)	19.8 (17.8-21.8)	15.2 (13.0-17.4)
90-99	7.6 (4.8-10.4)	22.7 (18.2-27.1)	22.3 (13.8-29.9)
Sex			
Male	3.3 (2.4-4.2)	12.0 (10.3-13.6)	11.5 (9.6-13.4)
Female	6.3 (5.3-7.4)	19.7 (18.0-21.4)	15.3 (13.2-17.3)
Race/ethnicity			
Non-Hispanic			
White	5.2 (4.4-6.0)	16.8 (15.5-18.1)	12.2 (10.7-13.6)
Black	3.5 (1.5-5.6)	15.3 (11.2-19.3)	24.7 (17.3-31.4)
Hispanic	5.0 (1.4-8.5)	9.8 (5.0-14.7)	20.7 (12.0-28.5)
Educational level			
Less than high school/GED	5.4 (4.2-6.5)	15.3 (13.5-17.2)	16.2 (13.2-19.0)
High school	4.9 (3.6-6.2)	16.6 (14.4-18.8)	11.5 (9.4-13.6)
Some college	5.4 (3.5-7.2)	18.7 (15.5-21.9)	10.4 (7.7-13.0)
College or above	3.9 (2.1-5.7)	16.6 (13.1-20.1)	9.8 (7.0-12.5)
Smoking status			
Never	7.5 (6.1-8.8)	20.7 (18.6-22.7)	17.3 (14.9-19.6)
Former	3.8 (2.9-4.7)	14.7 (13.0-16.4)	12.2 (10.3-14.1)
Current	0.7 (0.0-1.6)	6.3 (3.7-8.9)	5.2 (0.9-9.3)
Diabetes			
No	5.5 (4.7-6.4)	17.6 (16.2-19.0)	13.6 (12.1-15.2)
Yes	3.0 (1.7-4.3)	11.4 (9.0-13.7)	13.6 (10.3-16.7)
Hypertension			
No	5.7 (4.5-6.9)	18.5 (16.5-20.4)	15.8 (13.8-17.9)
Yes	4.6 (3.8-5.5)	14.9 (13.4-16.4)	11.9 (9.9-13.8)
Stroke			
No	5.1 (4.3-5.9)	15.5 (14.2-16.8)	11.3 (9.8-12.7)
Yes	5.0 (3.4-6.6)	20.4 (14.4-23.4)	24.4 (20.2-28.4)
Heart conditions			
No	5.9 (4.9-6.9)	17.8 (16.1-19.4)	14.6 (12.9-16.4)
Yes	3.9 (2.9-4.8)	14.6 (12.8-16.4)	11.9 (9.6-14.2)
US Census division^b^			
New England	2.5 (0.4-4.6)	13.0 (8.5-17.6)	13.8 (6.3-20.8)
Middle Atlantic	3.0 (1.5-4.6)	13.6 (10.5-16.8)	13.5 (9.5-17.4)
East North Central	5.7 (3.9-7.5)	18.1 (15.2-21.1)	12.6 (9.6-15.5)
West North Central	7.1 (4.4-9.9)	17.3 (13.3-21.3)	13.5 (9.3-17.5)
South Atlantic	5.4 (3.7-7.0)	16.2 (13.5-18.9)	13.3 (10.2-16.4)
East South Central	6.0 (2.4-9.6)	18.7 (12.8-24.6)	19.9 (11.2-27.7)
West South Central	4.0 (2.0-5.9)	13.0 (9.6-16.3)	16.6 (11.3-21.6)
Mountain	4.4 (1.5-7.4)	15.9 (10.6-21.1)	12.9 (6.7-18.6)
Pacific	6.0 (3.9-8.2)	19.9 (16.3-23.5)	11.6 (8.2-14.8)

Abbreviations: GED, general education development; PAF, population-attributable fraction.

^a^ The appearance of dementia anywhere on the death certificate.

^b^ New England (Connecticut, Maine, Massachusetts, New Hampshire, Rhode Island, and Vermont), Middle Atlantic (New York, New Jersey, and Pennsylvania), East North Central (Indiana, Illinois, Michigan, Ohio, and Wisconsin), West North Central (Iowa, Kansas, Minnesota, Missouri, Nebraska, North Dakota, and South Dakota), South Atlantic (Delaware; Washington, DC; Florida; Georgia; Maryland; North Carolina; South Carolina; Virginia; and West Virginia), East South Central (Alabama, Kentucky, Mississippi, and Tennessee), West South Central (Arkansas, Louisiana, Oklahoma, and Texas); Mountain (Arizona, Colorado, Idaho, New Mexico, Montana, Utah, Nevada, and Wyoming), Pacific (Alaska, California, Hawaii, Oregon, and Washington).

**Figure 3.**
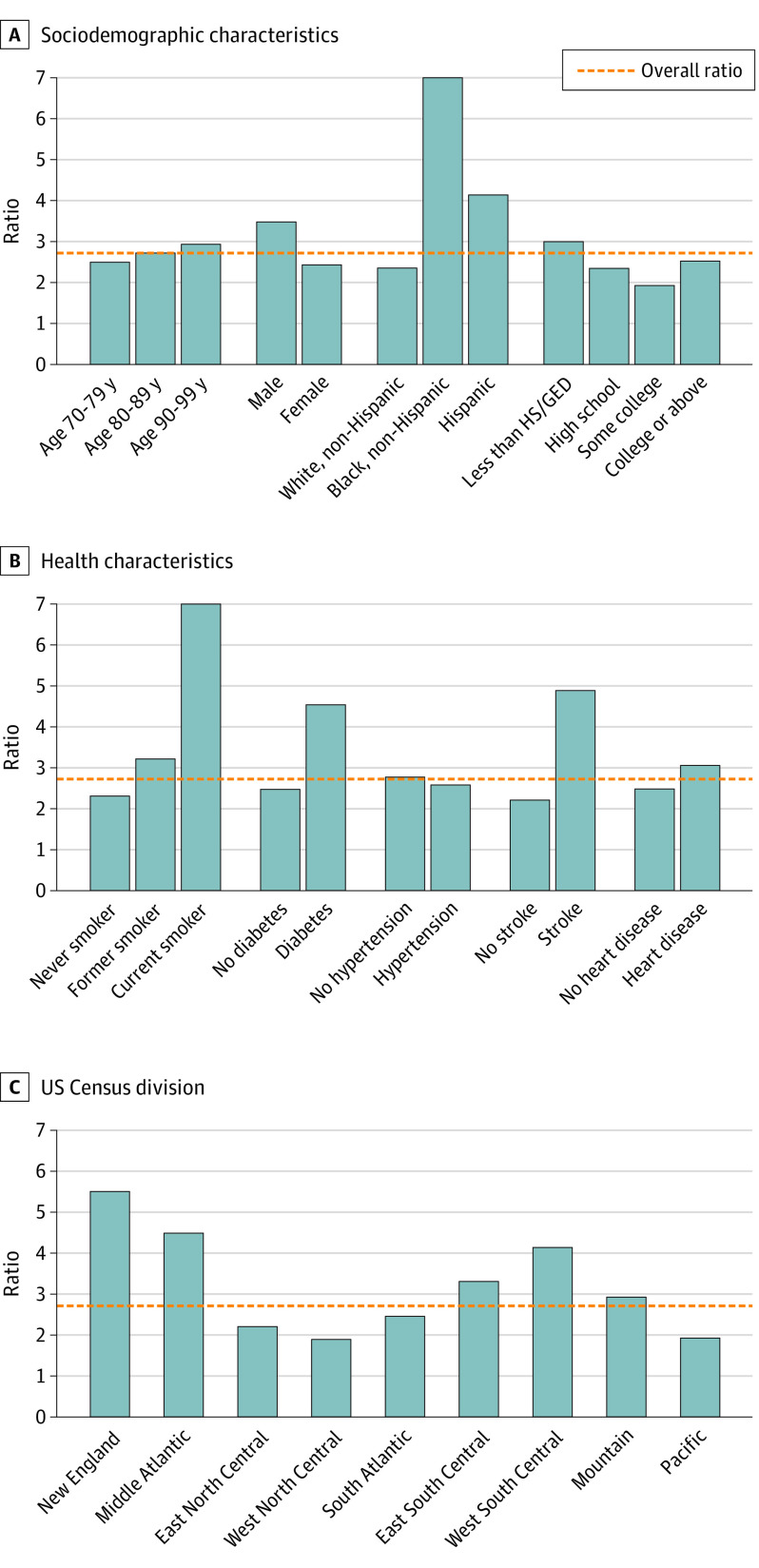
Ratio of Population-Attributable Fraction to Underlying Cause of Death Estimates HS/GED indicates high school/general educational development.

The extent to which underlying cause of death underestimated the mortality burden relative to the PAF varied by sociodemographic characteristics, health status, and geography (Figure 3). The extent of underestimation was greater among non-Hispanic Black and Hispanic participants compared with non-Hispanic White individuals (non-Hispanic Black: 7.1 times, Hispanic: 4.1 times, and non-Hispanic White: 2.3 times), in men compared with women (men: 3.5 times and women: 2.4 times), and in respondents with less than a high school education compared with those with high school or above (less than a high school education: 3.0 times, high school: 2.3 times, some college: 1.9 times, and college or above: 2.5 times). These findings were consistent across all 4 alternative classification algorithms, including modified Hurd, which was designed to increase sensitivity in studying disparities (eTable 3 in the Supplement).

Table 2 also presents the proportion of deaths in which dementia was mentioned anywhere on the death certificate. Considering any mention, the proportion of deaths associated with dementia increased to 16.4% (95% CI, 15.2%-17.6%), exceeding the burden estimated using the PAF. For CIND, although the HR was lower than for dementia, the prevalence was greater (24.7%), leading to a similar mortality burden at the population level (PAF, 10.2%; 95% CI, 8.3%-12.1%). Combining the PAF estimates for CIND and dementia (PAF*), the overall burden of cognitive impairment on mortality was an estimated 23.8%, which is 4.8 times the underlying cause of death estimate (eTable 4 in the Supplement).

## Discussion

In this nationally representative study of older US adults linked to mortality records, we found that approximately 13.6% of deaths were attributable to dementia over the period 2000-2009. This estimate was 2.7 times larger than vital statistics data on underlying cause of death. When considering deaths attributable to CIND as well, the disparity between the PAF and the underlying cause of death estimate increased to a factor of 4.8.

### Strengths and Limitations

One feature of the present study was the ability to compare survey- and vital statistics–based estimates of the contribution of dementia to mortality in the US population in a single source of data. One previous study used data that were limited to respondents from the greater Chicago area and did not attempt causal attribution of deaths to ADRD.^17^ Thus, as noted by the authors, their estimated dementia mortality burden of 32% for adults older than 65 years likely represents an upper boundary. A second study used a similar PAF approach to ours and estimated an even higher mortality burden of 37% among adults aged 75 to 84 years.^18^ However, the estimates of risk were calculated without adjustment for comorbidities using a nonprobability-based sample of respondents who agreed to postmortem brain donation. By using a single, nationally representative data source with internally consistent measures of exposure and outcome,^38^ our study may provide more valid estimates of the PAF and extent of underreporting than earlier studies that used community-based samples with unclear generalizability.

A second feature of the present study was our use of a PAF approach. Because people with dementia die of a combination of causes, calculating all deaths among people with dementia is likely to overestimate the dementia burden. A third feature of the present study was the additional examination of CIND in the population mortality burden of ADRD. Although the mortality risks associated with CIND are lower than those for dementia, CIND is more prevalent, such that its overall influence on the population is substantial.

This study also had limitations. First, we were not able to examine mortality associations separately for dementia subtypes, such as AD and vascular dementia. Second, although we used validated criteria derived from the Aging, Demographics, and Memory Study for assessing CIND and dementia status in the HRS, these classifications may be subject to measurement error. Third, the time lag between baseline cognitive assessment and follow-up for mortality could introduce misclassification if respondents who did not have dementia at baseline developed it later. We anticipate that this misclassification would have a downward bias on our results and a study design with shorter follow-up would yield even larger PAF estimates. Fourth, although use of PAF provides an alternative to calculating the mortality burden of dementia, the validity of PAF relies on several assumptions common to all observational studies, such as an absence of residual and unmeasured confounding. To reduce the risk of confounding in the present study, we adjusted for a set of sociodemographic, health, and geographic covariates that may be associated with dementia and risk of mortality. Fifth, estimates of mortality derived from the HRS could be biased by incomplete mortality ascertainment.However, a previous analysis suggested a close association between mortality rates estimated using the HRS and national vital statistics.^33^ Furthermore, any potential bias should not affect our estimates of underreporting because we compared survey- and vital statistics–based estimates calculated from within the same HRS sample.

## Conclusions

The findings of this study suggest that routine mortality statistics may underestimate the mortality burden associated with dementia by a factor of 2.7. Future research could examine the extent to which deaths attributable to dementia and underestimation of dementia as an underlying cause of death on death certificates might have changed over time. These results suggest that the mortality burden of dementia may be greater than recognized and highlight the importance of expanding access to population-based interventions focused on dementia prevention and care.

## Notes

Supplement. **eFigure 1.** Diagram of Inclusion/Exclusion Criteria **eFigure 2.** Kaplan Meier Survival Curves for Dementia and CIND Relative to No Cognitive Impairment **eTable 1.** Prevalence of Dementia and CIND for All Sample and Decedents, and by Population Subgroups Defined by Socio-Demographic Characteristics and Health Status **eTable 2.** Hazard Ratios From Cox Models Predicting Mortality With Sequential Covariate Adjustment **eTable 3.** PAF-Based Estimates of Deaths Attributable to Dementia Using Dichotomous Dementia Outcome and Alternative Specifications/Algorithms Across Socio-Demographic Characteristics **eTable 4.** Percentage of Deaths Attributable to Dementia and CIND Contrasting Underlying, any Mention, and PAF-Based Estimates
