# Supplementary material for: Estimates of the Association of Dementia With US Mortality Levels Using Linked Survey and Mortality Records
Source: JAMA Neurol. 2020 Aug 24;77(12):1–9. doi: 10.1001/jamaneurol.2020.2831 (PMC7445631; doi:10.1001/jamaneurol.2020.2831)
Supplement: Supplement. — eFigure 1. Diagram of Inclusion/Exclusion Criteria eFigure 2. Kaplan Meier Survival Curves for Dementia and CIND Relative to No Cognitive Impairment eTable 1. Prevalence of Dementia and CIND for All Sample and Decedents, and by Population Subgroups Defined by Socio-Demographic Characteristics and Health Status eTable 2. Hazard Ratios From Cox Models Predicting Mortality With Sequential Covariate Adjustment eTable 3. PAF-Based Estimates of Deaths Attributable to Dementia Using Dichotomous Dementia Outcome and Alternative Specifications/Algorithms Across Socio-Demographic Characteristics eTable 4. Percentage of Deaths Attributable to Dementia and CIND Contrasting Underlying, any Mention, and PAF-Based Estimates [file jamaneurol-e202831-s001.pdf]

Supplementary Online Content

Stokes AC, Weiss J, Lundberg DJ, et al. Estimates of the association of dementia with US mortality levels using linked survey and mortality records. *JAMA Neurol*. Published online August 24, 2020. doi:10.1001/jamaneurol.2020.2831

eFigure 1. Diagram of Inclusion/Exclusion Criteria

eFigure 2. Kaplan Meier Survival Curves by Cognitive Status

eTable 1. Prevalence of Dementia and CIND for All Sample and Decedents, and by Population Subgroups Defined by Socio-Demographic Characteristics and Health Status

eTable 2. Hazard Ratios From Cox Proportional Hazards Regression Models Predicting Mortality With Sequential Covariate Adjustment

eTable 3. PAF-Based Estimates of Deaths Attributable to Dementia Using Dichotomous Dementia Outcome and Alternative Specifications Across Socio-Demographic Characteristics

eTable 4. Percentage of Deaths Attributable to Dementia and CIND Contrasting Underlying, any Mention, and PAF-Based Estimates

This supplementary material has been provided by the authors to give readers additional information about their work.

eFigure 1. Diagram of inclusion/exclusion criteria

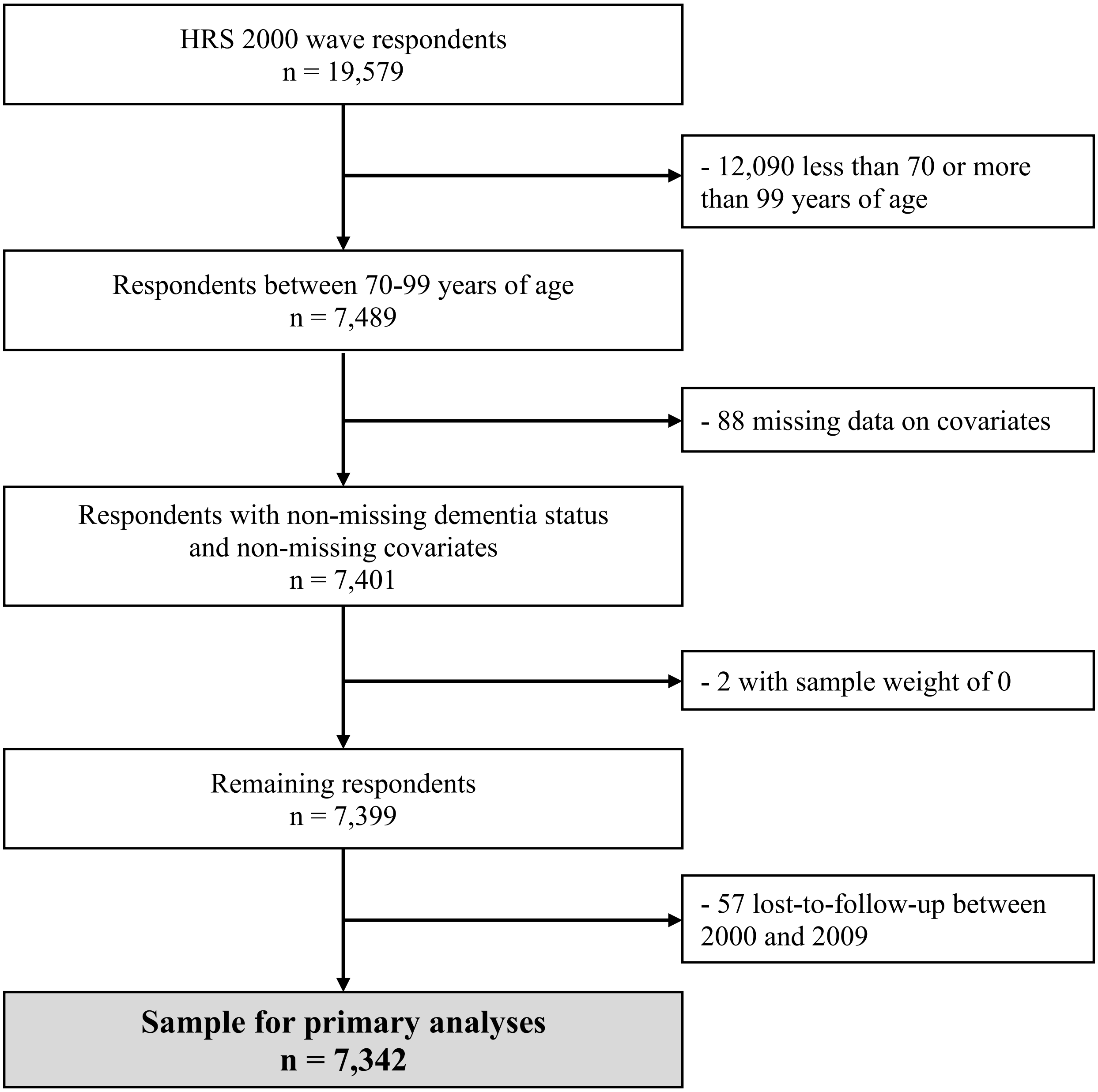

eFigure 2. Kaplan Meier survival curves by cognitive status[a]

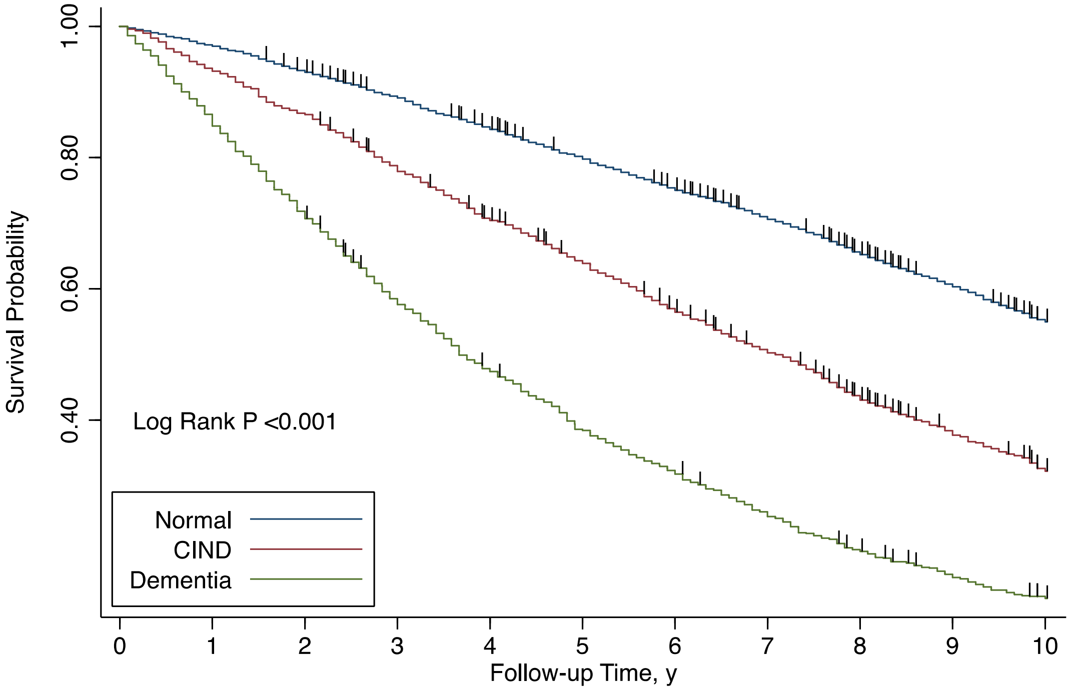

No. at risk

	0	1	2	3	4	5	6	7	8	9	10
Normal	4366	4243	4070	3873	3666	3454	3240	3022	2756	2490	2131
CIND	1843	1725	1599	1447	1296	1167	1030	907	770	655	541
Dementia	1133	981	814	654	534	430	360	285	219	172	131

a. Vertical bars on survival curves represent censored cases.

eTable 1. Prevalence of dementia and CIND for all sample and decedents, and by population subgroups defined by socio-demographic characteristics and health status

Characteristic	Complete Sample					
	Dementia			CIND		
	%	95% CI		%	95% CI	
All	14.3	13.5	15.2	24.7	23.6	25.7
Age category						
70-79	8.6	7.7	9.5	21.7	20.4	23.0
80-89	21.1	19.4	22.9	29.4	27.5	31.4
90-99	45.4	40.3	50.5	33.1	28.4	38.1
Sex						
Male	12.2	11.0	13.5	26.3	24.6	28.1
Female	15.7	14.6	16.9	23.6	22.3	25.0
Race/Ethnicity						
White, non-Hispanic	12.1	11.2	13.0	22.6	21.5	23.8
Black, non-Hispanic	31.0	27.6	34.6	37.9	34.2	41.8
Hispanic	22.0	18.2	26.3	39.0	34.1	44.1
Education						
Less than high school/GED	24.6	22.9	26.3	34.6	32.7	36.5
High school	9.6	8.4	11.0	22.2	20.4	24.2
Some college	8.7	7.2	10.6	18.8	16.6	21.2
College or above	5.4	4.1	7.0	12.2	10.3	14.4
Smoking Status						
Never	16.0	14.8	17.4	24.5	22.9	26.1
Former	13.2	12.0	14.5	24.5	23.0	26.1
Current	10.8	8.5	13.7	26.5	22.8	30.6
Diabetes						
No	13.7	12.9	14.7	23.7	22.5	24.8
Yes	17.4	15.2	19.8	30.2	27.4	33.2
Hypertension						
No	13.3	12.1	14.5	23.1	21.6	24.6
Yes	15.2	14.1	16.5	26.1	24.6	27.6
Stroke						
No	11.6	10.8	12.5	24.5	23.4	25.7
Yes	31.6	28.5	34.9	25.8	22.9	28.8
Heart conditions						
No	12.3	11.4	13.3	23.5	22.3	24.8
Yes	18.4	16.8	20.1	27.0	25.1	29.0
US census divisions						
New England	16.2	12.5	20.7	26.0	21.4	31.2
Middle Atlantic	13.3	11.1	15.7	22.0	19.4	24.9
East North Central	12.4	10.6	14.4	23.3	20.9	25.9
West North Central	12.8	10.2	15.9	23.9	20.6	27.6
South Atlantic	15.4	13.6	17.3	25.8	23.6	28.2
East South Central	18.9	14.8	23.9	24.6	20.0	29.9
West South Central	22.0	19.0	25.2	29.4	26.1	33.0
Mountain	11.2	8.0	15.5	25.5	21.0	30.7
Pacific	10.6	8.7	12.8	23.5	20.8	26.4

eTable 1. (Continued)

Characteristic	Decedents					
	Dementia			CIND		
	%	95% CI		%	95% CI	
All	22.4	21.0	23.8	29.3	27.8	30.9
Age category						
70-79	15.6	13.8	17.4	27.1	25.0	29.3
80-89	25.4	23.3	27.6	31.3	29.0	33.6
90-99	47.1	41.9	52.4	32.6	27.8	37.8
Sex						
Male	18.6	16.7	20.6	30.7	28.4	33.0
Female	25.1	23.3	27.1	28.4	26.4	30.4
Race/Ethnicity						
White, non-Hispanic	19.7	18.2	21.2	28.2	26.6	29.9
Black, non-Hispanic	42.1	37.3	47.0	36.5	31.8	41.4
Hispanic	33.0	26.8	39.9	40.7	34.0	47.8
Education						
Less than high school/GED	32.1	29.8	34.5	35.2	32.8	37.7
High school	16.9	14.7	19.4	27.6	25.0	30.5
Some college	15.5	12.7	18.8	25.6	22.2	29.4
College or above	11.7	9.0	15.1	18.2	15.0	22.0
Smoking Status						
Never	26.5	24.3	28.8	30.6	28.3	33.0
Former	20.3	18.5	22.3	28.2	26.2	30.4
Current	14.6	11.3	18.7	29.4	24.7	34.5
Diabetes						
No	22.2	20.8	23.8	28.7	27.1	30.4
Yes	23.0	20.0	26.2	31.9	28.5	35.5
Hypertension						
No	22.5	20.5	24.7	28.9	26.7	31.2
Yes	22.3	20.5	24.2	29.6	27.7	31.7
Stroke						
No	18.8	17.4	20.2	29.9	28.3	31.6
Yes	38.2	34.5	42.1	26.7	23.4	30.2
Heart conditions						
No	21.4	19.7	23.2	29.3	27.4	31.3
Yes	23.8	21.7	26.0	29.3	27.0	31.7
US census divisions						
New England	23.3	17.7	30.1	29.5	23.1	36.7
Middle Atlantic	21.3	17.7	25.3	27.1	23.3	31.4
East North Central	20.4	17.4	23.8	28.9	25.4	32.7
West North Central	21.2	16.9	26.3	29.0	24.3	34.3
South Atlantic	23.7	20.9	26.7	30.0	27.0	33.3
East South Central	28.9	22.3	36.5	29.0	22.3	36.6
West South Central	30.1	25.8	34.7	31.6	27.2	36.2
Mountain	18.1	12.9	24.9	26.6	20.6	33.6
Pacific	17.7	14.5	21.4	30.4	26.4	34.8

Abbreviations: GED, Graduate Equivalency Degree; CIND, Cognitive Impairment No Dementia

eTable 2. Hazard ratios from Cox proportional hazards regression models predicting mortality with sequential covariate adjustment

**Variables**	Model 1		Model 2		Model 3		Model 4	
	HR	95%CI	HR	95%CI	HR	95%CI	HR	95%CI
Cognitive function								
Normal (ref.)	1.00		1.00		1.00		1.00	
CIND	1.61	(1.49, 1.74)	1.61	(1.49, 1.75)	1.54	(1.41, 1.67)	1.53	(1.41, 1.67)
Dementia	2.71	(2.48, 2.97)	2.82	(2.55, 3.11)	2.52	(2.28, 2.80)	2.53	(2.28, 2.80)
Age	1.08	(1.08, 1.09)	1.08	(1.08, 1.09)	1.09	(1.09, 1.10)	1.09	(1.09, 1.10)
Sex								
Male (ref.)			1.00		1.00		1.00	
Female			0.73	(0.68, 0.78)	0.82	(0.76, 0.88)	0.82	(0.76, 0.88)
Race/ethnicity								
White, non-Hispanic (ref.)			1.00		1.00		1.00	
Black, non-Hispanic			0.84	(0.75, 0.94)	0.83	(0.74, 0.93)	0.82	(0.73, 0.93)
Hispanic			0.71	(0.60, 0.83)	0.77	(0.66, 0.90)	0.77	(0.66, 0.90)
Education								
High school (ref.)			1.00		1.00		1.00	
Less than high school/GED			1.01	(0.93, 1.10)	0.97	(0.89, 1.06)	0.98	(0.90, 1.06)
Some college			0.95	(0.86, 1.04)	0.94	(0.85, 1.04)	0.94	(0.85, 1.04)
College or above			0.89	(0.80, 0.99)	0.95	(0.85, 1.06)	0.95	(0.85, 1.06)

eTable 2. (Continued)

**Variables**	Model 1		Model 2		Model 3		Model 4	
	HR	95%CI	HR	95%CI	HR	95%CI	HR	95%CI
Smoking Status								
Never (ref.)					1.00		1.00	
Former					1.27	(1.18, 1.37)	1.27	(1.18, 1.37)
Current					2.11	(1.84, 2.42)	2.10	(1.83, 2.41)
Diabetes					1.51	(1.37, 1.65)	1.51	(1.37, 1.65)
Hypertension					1.15	(1.07, 1.23)	1.15	(1.08, 1.23)
Stroke					1.42	(1.29, 1.55)	1.42	(1.30, 1.56)
Heart diseases					1.42	(1.32, 1.52)	1.42	(1.32, 1.52)
US Census Division								
South Atlantic (ref.)							1.00	
New England							0.89	(0.75, 1.05)
Middle Atlantic							0.91	(0.80, 1.02)
East North Central							0.95	(0.85, 1.06)
West North Central							0.97	(0.85, 1.10)
East South Central							0.90	(0.75, 1.07)
West South Central							0.93	(0.81, 1.05)
Mountain							1.08	(0.92, 1.27)
Pacific							0.93	(0.83, 1.04)

* Coefficients adjusted for sampling weights.

eTable 3. PAF-based estimates of deaths attributable to dementia using dichotomous dementia outcome and alternative specifications across socio-demographic characteristics (n=6,615)

	Langa-Weir		Herzog-Wallace		Wu		Hurd		Modified Hurd	
	%	95% CI	%	95% CI	%	95% CI	%	95% CI	%	95% CI
All	10.2	8.7 , 11.6	6.3	5.3 , 7.3	9.4	8.0 , 10.8	16.1	14.3 , 17.9	9.4	8.2 , 10.6
Age groups										
70-79	7.7	6.0 , 9.5	4.3	3.1 , 5.6	5.5	4.0 , 7.0	9.6	7.8 , 11.4	4.7	3.5 , 6.0
80-89	11.8	9.6 , 13.9	7.9	6.4 , 9.5	12.0	9.8 , 14.2	19.9	16.7 , 23.0	13.0	11.0 , 15.0
90-99	17.3	9.7 , 24.2	11.7	5.9 , 17.1	20.5	10.4 , 29.4	28.0	14.1 , 39.7	21.3	13.0 , 28.8
Sex										
Male	8.4	6.5 , 10.2	4.3	2.9 , 5.6	5.5	3.9 , 7.1	13.1	10.7 , 15.5	6.5	4.9 , 8.1
Female	11.7	9.7 , 13.7	7.9	6.5 , 9.3	12.5	10.3 , 14.7	18.4	15.8 , 21.0	11.7	9.9 , 13.4
Race/Ethnicity										
White, non-Hispanic	9.3	7.9 , 10.7	6.0	5.0 , 7.0	9.2	7.7 , 10.6	15.8	13.9 , 17.7	8.9	7.6 , 10.2
Black, non-Hispanic	16.8	9.4 , 23.6	10.2	5.8 , 14.3	12.9	7.1 , 18.4	20.0	12.7 , 26.6	14.9	10.1 , 19.4
Hispanic	15.6	6.8 , 23.7	6.1	1.2 , 10.8	10.5	1.5 , 18.7	12.9	2.5 , 22.2	11.9	4.1 , 19.0
Education										
Less than high school/GED	12.3	9.4 , 15.2	7.2	5.2 , 9.1	12.0	9.2 , 14.8	18.5	15.1 , 21.7	11.6	9.3 , 13.8
High school	8.6	6.7 , 10.6	5.8	4.3 , 7.2	8.0	5.9 , 10.0	14.8	11.7 , 17.8	8.0	6.0 , 9.9
Some college	9.2	6.5 , 11.8	6.4	4.3 , 8.4	8.4	5.6 , 11.2	16.4	12.3 , 20.3	9.2	6.6 , 11.8
College or above	7.3	4.7 , 9.7	5.3	3.2 , 7.4	6.9	4.2 , 9.5	11.2	7.5 , 14.8	7.3	4.7 , 9.8
Smoking Status										
Never	12.3	9.9 , 14.6	8.4	6.7 , 10.1	13.3	10.7 , 15.7	20.7	17.6 , 23.8	12.5	10.4 , 14.7
Former	9.5	7.6 , 11.4	5.5	4.1 , 6.9	7.7	5.8 , 9.5	13.8	11.3 , 16.2	8.5	6.9 , 10.1
Current	3.4	-0.9 , 7.5	0.6	-2.1 , 3.2	1.3	-2.3 , 4.8	8.4	4.0 , 12.6	0.4	-2.6 , 3.4
Diabetes										
No	10.2	8.6 , 11.7	6.9	5.7 , 8.0	10.2	8.7 , 11.8	16.5	14.4 , 18.5	10.0	8.7 , 11.4
Yes	10.3	7.1 , 13.4	4.2	2.1 , 6.2	6.2	3.0 , 9.3	14.7	10.8 , 18.4	7.4	4.7 , 9.9
Hypertension										
No	12.5	10.5 , 14.6	7.9	6.4 , 9.5	12.3	10.2 , 14.3	19.5	16.8 , 22.1	10.8	8.9 , 12.6
Yes	8.4	6.4 , 10.3	5.2	3.8 , 6.5	7.4	5.5 , 9.4	13.8	11.3 , 16.1	8.5	6.8 , 10.1
Stroke										
No	8.3	6.9 , 9.7	5.4	4.4 , 6.4	7.7	6.3 , 9.2	14.3	12.3 , 16.1	7.5	6.2 , 8.7
Yes	19.3	15.0 , 23.3	10.8	7.5 , 13.9	17.4	13.0 , 21.7	24.9	19.7 , 29.7	18.6	14.9 , 22.3
Heart conditions										
No	11.2	9.4 , 12.9	7.3	6.0 , 8.6	10.4	8.7 , 12.1	17.6	15.3 , 19.9	10.5	9.0 , 12.0
Yes	8.7	6.5 , 11.0	5.0	3.4 , 6.6	7.9	5.4 , 10.3	13.8	10.8 , 16.6	8.0	5.9 , 10.0
US census divisions										
New England	12.2	5.1 , 18.7	4.7	-0.6 , 9.7	9.8	3.2 , 15.9	16.8	7.9 , 24.7	7.7	2.2 , 12.9
Middle Atlantic	11.6	7.8 , 15.2	7.8	5.0 , 10.5	11.0	7.3 , 14.5	17.8	12.9 , 22.5	10.3	6.9 , 13.6
East North Central	10.2	7.1 , 13.2	4.9	2.6 , 7.1	7.5	4.1 , 10.8	16.0	11.9 , 19.9	8.5	5.5 , 11.4
West North Central	11.0	6.9 , 15.0	8.0	4.7 , 11.2	11.9	7.6 , 16.0	18.3	12.6 , 23.7	10.5	7.0 , 13.9
South Atlantic	8.4	5.2 , 11.4	5.4	3.3 , 7.4	8.7	5.6 , 11.7	13.9	10.0 , 17.6	8.9	6.5 , 11.2
East South Central	15.9	7.0 , 23.9	12.0	6.1 , 17.5	16.8	8.5 , 24.4	20.1	9.1 , 29.9	14.4	7.5 , 20.7
West South Central	8.8	3.2 , 14.2	6.5	3.1 , 9.8	9.7	4.6 , 14.5	16.9	9.8 , 23.4	10.1	5.3 , 14.7
Mountain	9.8	3.8 , 15.5	6.5	2.5 , 10.3	9.8	4.5 , 14.8	15.4	8.4 , 21.8	10.0	4.5 , 15.2
Pacific	9.7	6.5 , 12.9	6.5	3.8 , 9.1	8.4	5.2 , 11.6	15.1	10.4 , 19.5	9.4	6.4 , 12.3

Abbreviations: PAF, Population Attributable Fraction; GED, Graduate Equivalency Degree

* Calculated using dichotomous dementia status across all 4 exposure classification algorithms as validated in Gianattasio et al., 2019

eTable 4. Percentage of deaths attributable to dementia and CIND contrasting underlying, any mention, and PAF-based estimates

	Underlying			Any Mention			PAF (Dementia)			PAF (CIND)			PAF (Combined)
Characteristic	%	95% CI		%	95% CI		%	95% CI		%	95% CI		%
All	5.0	4.3	5.8	16.4	15.2	17.6	13.6	12.2	15.0	10.2	8.3	12.1	23.8
Age groups													
70-79	4.1	3.1	5.0	12.2	10.7	13.8	10.2	8.4	12.0	9.8	7.1	12.5	20.0
80-89	5.6	4.4	6.7	19.8	17.8	21.8	15.2	13.0	17.4	10.3	7.4	13.2	25.5
90-99	7.6	4.8	10.4	22.7	18.2	27.1	22.3	13.8	29.9	6.2	-1.9	13.6	28.5
Sex													
Male	3.3	2.4	4.2	12.0	10.3	13.6	11.5	9.6	13.4	10.0	7.0	12.9	21.5
Female	6.3	5.3	7.4	19.7	18.0	21.4	15.3	13.2	17.3	10.2	7.7	12.6	25.5
Race/Ethnicity													
White, non-Hispanic	5.2	4.4	6.0	16.8	15.5	18.1	12.2	10.7	13.6	10.1	8.1	12.0	22.3
Black, non-Hispanic	3.5	1.5	5.6	15.3	11.2	19.3	24.7	17.3	31.4	11.1	2.9	18.6	35.8
Hispanic	5.0	1.4	8.5	9.8	5.0	14.7	20.7	12.0	28.5	10.9	-3.4	23.2	31.6
Education													
Less than high school/GED	5.4	4.2	6.5	15.3	13.5	17.2	16.2	13.2	19.0	9.0	5.3	12.6	25.2
High school	4.9	3.6	6.2	16.6	14.4	18.8	11.5	9.4	13.6	9.7	6.3	13.0	21.2
Some college	5.4	3.5	7.2	18.7	15.5	21.9	10.4	7.7	13.0	11.0	6.8	15.0	21.4
College or above	3.9	2.1	5.7	16.6	13.1	20.1	9.8	7.0	12.5	6.4	2.7	9.9	16.2
Smoking Status													
Never	7.5	6.1	8.8	20.7	18.6	22.7	17.3	14.9	19.6	13.3	10.3	16.2	30.6
Former	3.8	2.9	4.7	14.7	13.0	16.4	12.2	10.3	14.1	8.1	5.4	10.7	20.3
Current	0.7	0.0	1.6	6.3	3.7	8.9	5.2	0.9	9.3	7.3	0.6	13.4	12.5
Diabetes													
No	5.5	4.7	6.4	17.6	16.2	19.0	13.6	12.1	15.2	10.4	8.3	12.4	24.0
Yes	3.0	1.7	4.3	11.4	9.0	13.7	13.6	10.3	16.7	9.3	4.6	13.7	22.9
Hypertension													
No	5.7	4.5	6.9	18.5	16.5	20.4	15.8	13.8	17.9	11.3	8.5	14.1	27.1
Yes	4.6	3.8	5.5	14.9	13.4	16.4	11.9	9.9	13.8	9.1	6.4	11.7	21.0
Stroke													
No	5.1	4.3	5.9	15.5	14.2	16.8	11.3	9.8	12.7	10.2	8.1	12.3	21.5
Yes	5.0	3.4	6.6	20.4	14.4	23.4	24.4	20.2	28.4	9.6	5.4	13.6	34.0

Heart conditions													
No	5.9	4.9	6.9	17.8	16.1	19.4	14.6	12.9	16.4	11.4	9.0	13.8	26.0
Yes	3.9	2.9	4.8	14.6	12.8	16.4	11.9	9.6	14.2	8.0	9.6	14.2	19.9
US census divisions													
New England	2.5	0.4	4.6	13.0	8.5	17.6	13.8	6.3	20.8	5.6	-3.8	14.2	19.4
Middle Atlantic	3.0	1.5	4.6	13.6	10.5	16.8	13.5	9.5	17.4	8.0	2.5	13.3	21.5
East North Central	5.7	3.9	7.5	18.1	15.2	21.1	12.6	9.6	15.5	12.2	7.6	16.5	24.8
West North Central	7.1	4.4	9.9	17.3	13.3	21.3	13.5	9.3	17.5	9.9	3.4	16.0	23.4
South Atlantic	5.4	3.7	7.0	16.2	13.5	18.9	13.3	10.2	16.4	9.7	5.4	13.7	23.0
East South Central	6.0	2.4	9.6	18.7	12.8	24.6	19.9	11.2	27.7	13.8	4.0	22.6	33.7
West South Central	4.0	2.0	5.9	13.0	9.6	16.3	16.6	11.3	21.6	11.1	4.9	16.9	27.7
Mountain	4.4	1.5	7.4	15.9	10.6	21.1	12.9	6.7	18.6	5.3	-3.7	13.6	18.2
Pacific	6.0	3.9	8.2	19.9	16.3	23.5	11.6	8.2	14.8	11.2	6.2	16.0	22.8

Abbreviations: PAF, Population Attributable Fraction; GED, Graduate Equivalency Degree; CIND, Cognitive Impairment No Dementia. “Any mention” refers to the appearance of dementia as either the underlying or a contributing cause of death on the death certificate.
